# CETP Expression in Bone-Marrow-Derived Cells Reduces the Inflammatory Features of Atherosclerosis in Hypercholesterolemic Mice

**DOI:** 10.3390/biom13101556

**Published:** 2023-10-22

**Authors:** Thiago Rentz, Gabriel G. Dorighello, Renata R. dos Santos, Lohanna M. Barreto, Israelle N. Freitas, Carolina M. Lazaro, Daniela S. Razolli, Patricia M. Cazita, Helena C. F. Oliveira

**Affiliations:** 1 Department of Structural and Functional Biology, Institute of Biology, State University of Campinas, Campinas 13083-862, SP, Brazil; rentzthiago@gmail.com (T.R.); gabrieldorighello@gmail.com (G.G.D.); lomonali.nutri@gmail.com (L.M.B.); israellenettof@gmail.com (I.N.F.); carolinamakie@gmail.com (C.M.L.); 2 Division of Radiotherapy, Medical School Hospital, Faculty of Medical Sciences, State University of Campinas, Campinas 13083-887, SP, Brazil; renata.fisicamedica@gmail.com; 3 Obesity and Comorbidities Research Center, State University of Campinas, Campinas 13083-864, SP, Brazil; danirazolli@yahoo.com.br; 4 Laboratório de Lípides (LIM10), Hospital das Clínicas HCFMUSP, Faculdade de Medicina, Universidade de Sao Paulo, Sao Paulo 01246-903, SP, Brazil; p.cazita@hc.fm.usp.br

**Keywords:** CETP, atherosclerosis, inflammation, macrophages, nicotinamide nucleotide transhydrogenase, LDL receptor knockout mice

## Abstract

CETP activity reduces plasma HDL-cholesterol concentrations, a correlate of an increased risk of atherosclerotic events. However, our recent findings suggest that CETP expression in macrophages promotes an intracellular antioxidant state, reduces free cholesterol accumulation and phagocytosis, and attenuates pro-inflammatory gene expression. To determine whether CETP expression in macrophages affects atherosclerosis development, we transplanted bone marrow from transgenic mice expressing simian CETP or non-expressing littermates into hypercholesterolemic LDL-receptor-deficient mice. The CETP expression did not change the lipid-stained lesion areas but decreased the macrophage content (CD68), neutrophil accumulation (LY6G), and TNF-α aorta content of young male transplanted mice and decreased LY6G, TNF-α, iNOS, and nitrotyrosine (3-NT) in aged female transplanted mice. These findings suggest that CETP expression in bone-marrow-derived cells reduces the inflammatory features of atherosclerosis. These novel mechanistic observations may help to explain the failure of CETP inhibitors in reducing atherosclerotic events in humans.

## 1. Introduction

Cardiovascular diseases (CVD) remain the number one cause of death globally, killing about 18 million people every year [1,2]. Atherosclerosis is the dominant process in CVD and is characterized by a complex condition involving multiple factors related to the disruption of the cholesterol metabolism, inflammation, and redox homeostasis [2,3,4]. These events develop slowly and progressively and are linked to disturbances in low-density lipoprotein (LDL) physiology and LDL interaction with arterial wall cells, mainly with macrophages [5].

Macrophages act as immune sentinels, responding quickly to micro-environmental signals and changing their phenotype to develop specific functions such as inducing or resolving local inflammation [6]. During the early steps of atherogenesis, macrophages derived from circulating monocytes become lipid-laden foam cells by taking up chemically modified LDL trapped in the subendothelial space. These sequestered lipoproteins can induce endothelial dysfunction and promote the recruitment of additional blood-borne monocytes to the lesion site. Upon differentiating into macrophages, these cells internalize modified LDL and accumulate intracellular cholesterol, becoming pro-inflammatory and eventually undergoing cell death, forming a necrotic core within the lesion [2,3,6,7,8]. The monocyte-derived macrophages present in the atherosclerotic lesion are produced in the bone marrow [5], and macrophage accumulation in the established mouse atheroma may arise from local replication [9].

To investigate the impact of macrophages in atherosclerosis, bone marrow transplantation in mice offers a powerful and effective strategy. This approach allows for the generation of chimeric animals with a hematopoietic compartment carrying the donor’s genetic background, which enables the identification of the role of the genetic profile of these cells in the development of atherosclerosis [10]. Consistent evidence has shown that the reconstitution of *Apoe*^−/−^ mice with wild-type bone marrow cells results in protection from high lipid levels and atherosclerosis, and, conversely, wild-type mice reconstitution with *Apoe*^−/−^ bone marrow cells exacerbates atherosclerosis [11,12]. In addition, *Ldlr*^−/−^ mice exhibited increased atherosclerosis after transplantation with ABCA1-deficient or ABCA1 + SR-B1-deficient bone marrow, proteins that are involved in the efflux of membrane cholesterol from macrophages [13,14].

CETP expression and plasma activity may modulate atherosclerosis, and its role is likely highly to be dependent on metabolic context and genetic background. Most human and rabbit studies have suggested that CETP expression or activity promotes atherosclerosis progression, mainly through its effect of increasing non-HDL lipoproteins and decreasing HDL-cholesterol plasma levels. On the other hand, experimental evidence, mainly from genetically modified mice, supports the concept that CETP may protect against atherosclerosis when the LDL receptor function is preserved [15]. An important novel function of CETP that may be relevant for atherogenesis is its putative modulatory role on inflammation. Whole-body CETP expression protects mice from mortality in sepsis models [16]. Additionally, the survival rate of patients with sepsis correlates with the plasma CETP concentration [17]. However, contrary results have recently been reported in mice and humans [18,19]. These discrepancies are not surprising, since, in vivo, it is quite difficult to discriminate between the effects of the reciprocal changes in CETP and HDL concentrations on susceptibility or protection against acute inflammation.

Previous studies have demonstrated that CETP-expressing mice that are hypercholesterolemic due to *Apoe* or *Ldlr* gene deficiency develop more atherosclerosis than CETP non-expressing mice [20]. Moreover, female *Ldlr*^−/−^ mice transplanted with CETP-expressing bone marrow from transgenic mice exhibited increased atherosclerosis [21]. However, it is noteworthy that these experimental groups lacked the nicotinamide nucleotide transhydrogenase (*Nnt*) gene (C57BL/6J background), which encodes a mitochondrial enzyme that plays a critical role in the maintenance of cell redox [22], inflammatory [23] and cholesterol homeostasis [22], and predisposes to metabolic disturbances [24]. Indeed, our recent findings have shown that the transplantation of *Ldlr*^−/−^ mice (*Nnt* deficient) with *Nnt*-preserved bone marrow (C57BL/6JUnib) significantly reduced atherosclerosis development [22].

Therefore, in light of these findings, we propose that CETP-expressing macrophages with preserved *Nnt* expression (C57BL/6JUnib) offer a promising and beneficial strategy for modulating atherosclerosis development in the *Ldlr*^−/−^ background. Considering that atherosclerosis advances markedly with aging [25], is more severe in the female than the male *Ldlr*^−/−^ mouse model [25,26], and that CETP has sex-dependent effects on endothelial function [27], it is of interest to confirm research outcomes in more than one context. Thus, in this work, we tested the effects of CETP-expressing bone-marrow-derived cells in two distinct models of atherosclerosis: young male and aged female *Ldlr*^−/−^ mice. We found similar anti-inflammatory effects of CETP in the lesions of both mice models.

## 2. Materials and Methods

### 2.1. Mice

All the experimental protocols were performed in accordance with the National Council for Animal Experimentation Control (CONCEA) and were approved by the Ethics Committee on Animal Experimentation of the State University of Campinas (CEUA/UNICAMP #3814-1(A)/2017, #5270-1/2019). Mice lacking the LDL receptor gene, *Ldlr*^−/−^ (B6.129S7-Ldlrtm1Her/J), were purchased from The Jackson Laboratory in 2009 and maintained in the Multidisciplinary Center for Biological Investigation on Laboratory Animal Science (CEMIB/UNICAMP). Mice overexpressing simian CETP (sCETP) (Tg [CETP] UCTP20Pnu/J) were purchased from the Jackson Laboratory in 2013 and crossbred with C57BL/6JUnib (RRID:MGI:7264953) mice for more than 7 generations. The C57BL/6JUnib colony was originally supplied by the Zentralinstitut für Versuchstierzucht (ZfV) (Hannover, Germany) in 1987, which had previously been obtained from the Jackson Laboratory before the spontaneous deletion of the *Nnt* gene in their colony [28]. Simian CETP-expressing mice were screened with PCR using tail tip genomic DNA according to the Jackson Laboratory protocol. All the animal work was performed in the Lipid Metabolism Laboratory, Dept of Structural and Functional Biology, Institute of Biology, State University of Campinas. The mice were kept under standard laboratory conditions (at 22 ± 1 °C and 12 h light/dark cycle) in a local (conventional) animal facility, in individually ventilated cages (3–4 mice/cage) with free access to filtered tap water and a regular rodent AIN93 m diet (standard laboratory rodent chow diet, Nuvital CR1, Colombo, PR, Brazil). Two independent transplantation studies were performed using the following recipient mice: (1) male *Ldlr*^−/−^ mice of 7 weeks of age (n = 12/group) and (2) female *Ldlr*^−/−^ mice of 15–18 months of age (n = 7–11/group). The mice were allocated to receive bone marrow transplants from either sCETP transgenic or non-transgenic littermates as donors, while ensuring the appropriate matching of male donors to male recipients and female donors to female recipients. After the bone marrow transplantation, the mice had free access to sterile water and a sterile high-fat (22 g%) and high-cholesterol diet (0.15 g%) (Supplementary Table S1) for 8 weeks. For the terminal experiments, the mice were anesthetized with xylazine/ketamine (10 and 50 mg/Kg, respectively), ip, followed by exsanguination through the retro-orbital plexus for plasma analyses. The hearts were perfused and excised for the atherosclerosis analyses.

### 2.2. Irradiation and Bone Marrow Transplantation

Seven-week-old male and 15–18-month-old female *Ldlr*^−/−^ mice were exposed to a single 8.0 Gy total-body irradiation using a 6 MV linear accelerator (Varian Clinac 2100C, Medical Equipment Dynamics, New Bedford, MA). Immediately after irradiation, the animals were injected via the tail vein with 5.0 × 10^6^ bone marrow (BM) cells freshly collected from male or female donor mice, including both sCETP transgenic and non-transgenic mice, which were used for the bone marrow transplants. The BM cells were aseptically harvested by flushing the donor femurs and tibias with Dulbecco’s PBS containing 2% fetal bovine serum. The samples were filtered through a 40 µm nylon mesh and centrifuged at room temperature for 10 min at 300 g. The cells were suspended in 200 μL of Dulbecco’s PBS supplemented with 2% fetal bovine serum, at a concentration of 5.0 × 10^6^ viable nucleated cells per injection into the mouse tail vein. Subsequently, the mice underwent a one-week recovery period housed in autoclaved ventilated cages. The mice were treated with antibiotics (0.2 mg of trimethoprim and 1.0 mg/mL of sulfamethoxazole) in their drinking water for 4 days before and 7 days after the BM transplantation. The transplanted mice were subsequently placed on a high-fat and high-cholesterol diet for the following 8 weeks (Supplementary Table S1).

### 2.3. Plasma Lipids

Non-fasting plasma lipids were determined using enzymatic-colorimetric assays (Randox Laboratories Ltd., Crumlin, Northern Ireland) according to the manufacturer’s instructions. 

### 2.4. Aortic Root Lesion Area

The mice hearts were perfused in situ with phosphate-buffered saline (PBS) followed by 4% PBS-buffered formaldehyde. The hearts were then excised and embedded in Tissue-Tek^®^ OCT compound (Sakura Inc., Torrance, CA, USA) and frozen at −80 °C. The tip of the heart (ventricle) was removed with a surgical knife and serial sections of 60 µm were cut and discarded until the visualization of the aortic sinus leaflets. Then, sections were reduced to a 10 µm thickness and cut along a 480 µm aorta length. The sections were stained with Oil Red O. The red areas of the lesions were calculated as the sum of the lipid-stained lesions (1 section every 120 µm along the aorta length). The lipid-stained lesions were quantified using ImageJ software, version 1.51s (U.S. National Institutes of Health, Bethesda, MD, USA, http://imagej.nih.gov/ij). 

### 2.5. Lesion Area Immunofluorescence Staining

The same procedure for cryosections was employed in additional hearts for immunofluorescence staining. The cryosections were blocked with 10% bovine serum albumin (BSA) and then incubated for 3 h at 22 °C or overnight at 4 °C with the following primary antibodies: CD68 antibody (1:200; Bio-Rad, Hercules, CA, USA), purified anti-mouse Ly-6G antibody (1:50, Biolegend, San Diego, CA, USA), anti-TNF-α antibody (1:50, Abcam, Waltham, MA, USA), biotinylated nitrotyrosine (3-NT) (1:100; Cayman Chemical, Ann Arbor, MI, USA), anti-DNA/RNA damage antibody, epitope 8-oxo-7,8-dihydro-2′-deoxyguanosine (1:50, Abcam), and anti-iNOS (1:500, Thermo Fisher, Waltham, MA, USA). The sections were washed and incubated with fluorescent-labeled secondary antibody Alexa Fluor-conjugated (Invitrogen, Walthan, MA, USA). The nuclei were counterstained with DAPI for 10 min. The sections were mounted with Vectashield medium, microscopic images of the aortic lesions (objective lenses 10×) were digitalized, and morphometric measurements were calculated as described for Oil red O. ImageJ software, version 1.51s (U.S. National Institutes of Health, Bethesda, MD, USA, http://imagej.nih.gov/ij) was used for all the quantifications. 

### 2.6. Real Time PCR

The thoracic aortas’ RNA was extracted with the RNeasy kit (#7400, Qiagen, Hilden, Germany), then 1 µg of purified RNA was used to synthesize the cDNA (High-Capacity cDNA reverse transcription kit, Applied Biosystems, Foster City, CA, USA). Relative quantification was performed using the step-one real-time PCR system (Applied Biosystems, Waltham, MA, USA). The primers were designed and tested against the *Mus musculus* genome (GenBank, National Center for Biotechnology Information, U. S. National Institutes of Health, USA, https://www.ncbi.nlm.nih.gov/genbank/). The relative quantities of the target transcripts were calculated from duplicate samples (ΔΔCT) and normalized against the endogenous control 36B4 (acidic ribosomal phosphoprotein P0). The primer sequences are shown in the Supplementary Table S2. 

### 2.7. Statistical Analysis

Data are presented as the mean ± the standard error (SE) and individual experimental data. The number of mice (n) is stated in the figure legends. Statistical differences were evaluated using the Student’s *t*-Test. The animal sample size (n) for each experiment was chosen based on the literature documentation of similar studies. Significance was accepted at the level of *p* ≤ 0.05.

## 3. Results

### 3.1. Efficacy of Transplantation and Biometric and Plasma Data

**Figure 1:**
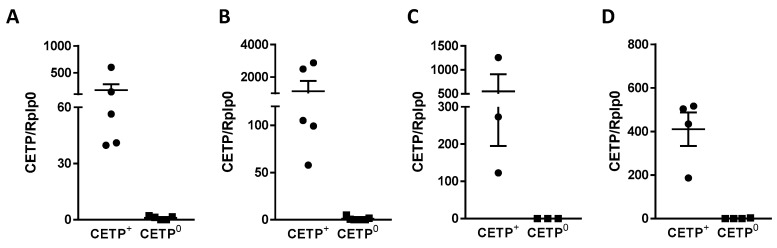
CETP mRNA relative expression in the aorta and spleen of recipient *Ldlr*^−/−^ mice. CETP mRNA expression in the aorta (**A**,**C**) and spleen (**B**,**D**) of young male (**A**,**B**) and aged female (**C**,**D**) *Ldlr*^−/−^ transplanted mice that received bone marrow from CETP transgenic (CETP^+^) or non-transgenic (CETP^0^) donor mice. Internal control: acidic ribosomal phosphoprotein P0 (Rplp0).

To confirm the efficacy of the transplantations, we measured the expression of CETP mRNA in the aorta and in a macrophage-rich tissue, the spleen, of the recipient *Ldlr*^−/−^ mice. Both tissues of both atherosclerosis models showed CETP mRNA expression in the CETP-positive (CETP^+^) bone marrow (BM) recipients compared to the CETP-null (CETP^0^) BM recipients. These data are shown in Figure 1.

The biometric data and plasma lipids after the BM transplantation are shown in Table 1. Young male *Ldlr*^−/−^ recipients of CETP-expressing (CETP^+^) BM showed increased plasma total cholesterol levels and decreased adipose tissue. These changes were not observed in the aged female *Ldlr*^−/−^ recipients of CETP^+^ BM.

### 3.2. Atherosclerosis Features

**Figure 2:**
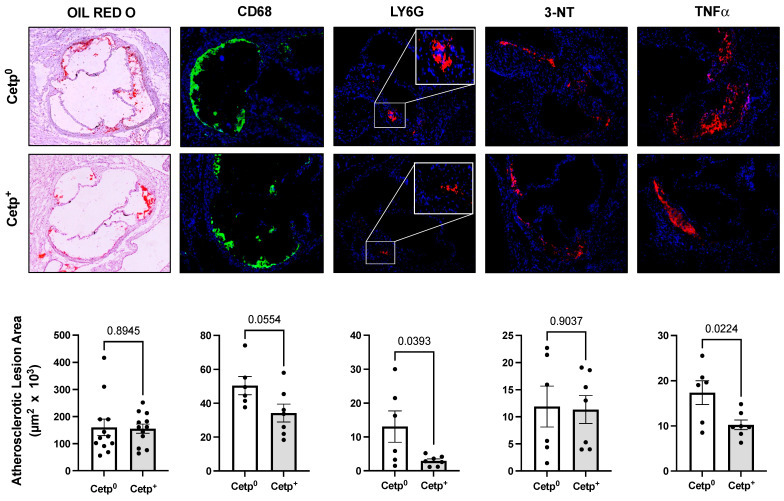
Aorta root atherosclerotic lesions of young male *Ldlr*^−/−^ mice transplanted with bone marrow from CETP-expressing (CETP^+^) or non-expressing (CETP^0^) mice after 8 weeks on a high-fat and high-cholesterol diet. The upper and middle panels show representative images of stained aortic root sections and the bottom panels show quantification of positive areas. From left to right: lipid staining with oil red O, immunostaining for macrophages (CD68), neutrophils (Ly6G), nitrotyrosine (3-NT), and TNF-α. Data are mean ± SE. Numbers above the bars are *p* values by Student *t* test.

**Figure 3:**
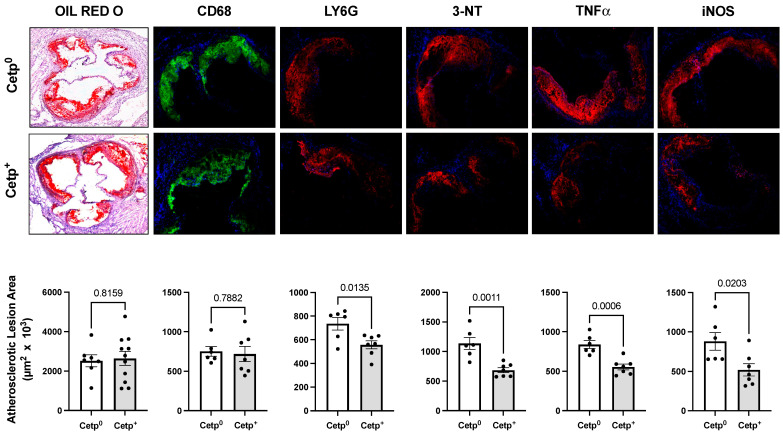
Aorta root atherosclerotic lesions of aged female *Ldlr*^−/−^ mice transplanted with bone marrow from CETP-expressing (CETP^+^) or non-expressing (CETP^0^) mice after 8 weeks on a high-fat and high-cholesterol diet. The upper and middle panels show representative images of stained aortic root sections and the bottom panels show quantification of positive areas. From left to right: lipid staining with oil red O, immunostaining for macrophages (CD68), neutrophils (Ly6G), nitrotyrosine (3-NT), TNF-α, and inducible nitric oxide synthase (iNOS). Data are mean ± SE. Numbers above the bars are *p* values by Student *t* test.

To quantify and characterize the atherosclerotic lesions in the transplanted mice, their aorta roots were stained with Oil Red O (lipid content) and immunostained for CD68 (marker of macrophages and monocytes), Ly6G (marker of neutrophils), TNF-α (classic inflammatory marker), 3-nitrotyrosine (3-NT, marker of nitro-oxidative stress), 8-oxo-7,8-dihydro-2′-deoxyguanosine (8-Oxo-dG, marker of DNA and RNA oxidative damage), and iNOS (marker of M1 pro-inflammatory type of mouse macrophage) (Figure 2 and Figure 3). In the young male *Ldlr*^−/−^ recipient mice, the CETP-expressing BM (CETP^+^) did not change the lipid-stained lesion areas compared to the CETP null BM (CETP^0^) recipient mice (Figure 2). However, the neutrophils (LY6G) and TNF-α content in the recipients of CETP^+^ BM were significantly reduced, and the macrophage (CD68) content also tended to be reduced in the CETP^+^ BM recipients (*p* = 0.055). The oxidized protein (3-NT, Figure 2) and nucleic acid (8-Oxo-dG, not shown) levels did not differ between the young male *Ldlr*^−/−^ recipient mice groups.

Since the observed lesion sizes in the young male mice were small (~2 × 10^5^ μm^2^), to confirm and expand these findings, we studied another independent group of recipient mice with more advanced atherosclerosis, namely, aged female *Ldlr*^−/−^ (15–18 months of age, Figure 3). The lipid (oil red) and macrophage (CD68) contents did not differ between the recipient groups. However, inflammatory markers (LY6G, TNF-α, and iNOS) and the nitroxidation of proteins (3-NT) were markedly decreased in the CETP^+^ BM aged female *Ldlr*^−/−^ recipient mice (Figure 3).

### 3.3. Aortic Gene Expression

**Figure 4:**
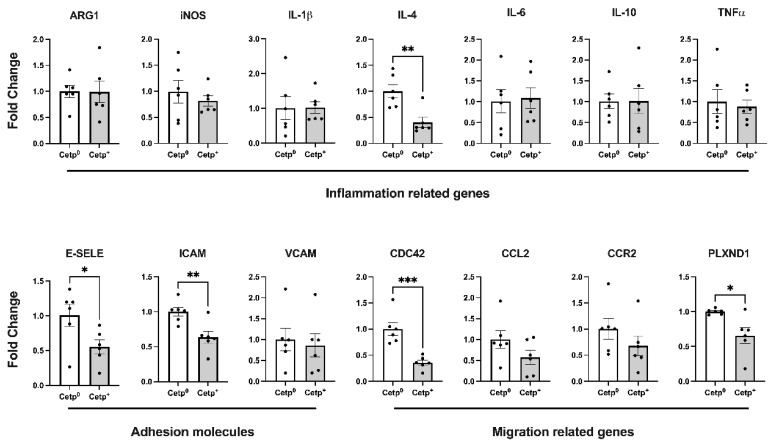
Gene expression in thoracic aortas of young male *Ldlr*^−/−^ mice transplanted with bone marrow from CETP-expressing (CETP^+^) or non-expressing (CETP^0^) mice. Upper panels show pro- and anti-inflammatory related genes; Bottom panels show adhesion molecule genes encoding E-Selectin, ICAM, and VCAM, and migration-related genes encoding CDC42, CCL2, CCR2, and PLXND1 proteins. Data are mean ± SE. * *p* < 0.05, ** *p* < 0.01, and *** *p* < 0.001 by Student *t* test.

We also evaluated, in the thoracic aorta segment, the expressions of the genes related to inflammation, adhesion, and the migration of cells (Figure 4). Except for IL-4, all the classical inflammation-related genes (IL-1β, IL-6, IL-10, TNF-α, iNOS, and ARG-1) were not differentially expressed in this segment of the aortas in the recipients of the CETP^+^ and CETP^0^ BM cells. However, the expressions of the adhesion molecules E-selectin and ICAM-1, as well as the expressions of the migration-related genes CDC42 (cell division cycle 42) and PLXND1 (Plexin D1), were decreased significantly in the aortas of the recipients of the CETP^+^ BM cells (Figure 4).

## 4. Discussion

CETP has a well-characterized action on determining fluxes in cholesterol among plasma lipoproteins, resulting in reduction in HDL-cholesterol that may be accompanied (or not) by increased non-HDL-cholesterol. Thus, CETP inhibition has been pursued as an anti-atherogenic target. However, its impact on atherosclerosis development is controversial, basically because it is largely dependent on diverse genetic and metabolic settings, particularly regarding the functionality of the LDL receptor pathway. Over the last decades, experimental atherosclerosis has been studied in C57BL/6J background mouse models (*Ldlr*^−/−^ and *Apoe*^−/−^), strains that also carry a spontaneous deletion of the mitochondrial antioxidant enzyme encoding *Nnt* gene. In this background, CETP is indeed atherogenic [20,21,29]. The Nnt enzyme, first characterized by Rydstrom and colleagues [30], has a pivotal role in determining cell redox state and susceptibility to metabolic diseases [22,24,28,30]. Because of its antioxidant action, *Nnt* expression in macrophages has proven anti-inflammatory [22,23] and anti-atherogenic effects [22]. Therefore, this study re-evaluated the role of CETP expression in macrophages with a preserved expression of *Nnt* in the development of experimental atherosclerosis. In fact, we found no differences in the lipid-stained lesion areas; however, CETP expression in bone-marrow-derived cells reduced the inflammatory features of atherosclerosis in the recipient mice, independently of plasma cholesterol levels. The results of the present study showed increased plasma cholesterol in young males and no changes in aged females transplanted with CETP-expressing bone marrow. These differential responses of blood cholesterol in CETP-transplanted mice are not surprising, since CETP activity varies according to metabolic contexts, including genetic background, diet, sex hormones, and age [15,27,31]. Thus, both results in these two models suggested that the observed CETP anti-inflammatory effects were independent of the plasma total cholesterol levels. Interestingly, decreased adipose tissue was observed in the young male recipients of CETP-expressing bone marrow. This was expected, since we previously reported an attenuation of adiposity in mice expressing CETP, in both young males and females [32]. The fact that this result was not observed in the aged females transplanted with CETP bone marrow may thus be related to the aging process. 

An original finding that deserves attention is the reduced content of neutrophils in the aortic lesions of the *Ldlr*^−/−^ mice expressing CETP in their bone-marrow-derived cells. Previous studies have shown that neutrophil depletion reduces the burden of atherosclerotic lesion in *Ldlr*^−/−^ mice, while hypercholesterolemia alone induces neutrophilia and accelerates atherogenesis [33]. In addition, the use of antibodies against Ly6G, a neutrophil-specific antigen, indicates that these cells are more abundant in regions of intense inflammatory activity in both *Ldlr*^−/−^ and *Apoe*^−/−^ mice models [33,34]. It is important to recall that the increase in circulating neutrophils and their accumulation occurred concomitantly with the beginning of a high-fat diet, contributing to increase lesion sizes [33]. Human data have shown that the content of neutrophils is associated with atherosclerotic plaque progression and instability [35]. Mechanistically, neutrophils exert pro-atherogenic effects via the production of reactive oxygen species and the release of granular proteins such as α-defensins, azurocidin, and LL-37, proteins previously localized in atherosclerotic plaques [36]. Neutrophils eventually undergo apoptosis and promote local inflammation. Generally, the apoptosis of neutrophils leads to DNA release and the formation of neutrophil extracellular traps (NETs), which are associated with the progression of atherosclerosis [37,38,39,40]. In this context, CETP’s anti-inflammatory properties likely attenuated the downstream effects of neutrophil accumulation. The effects of the CETP-expressing BM on reducing aortic neutrophils may have resulted from the reduction in TNF-α (and likely other pro-inflammatory cytokines and chemokines) in the lesion microenvironment. The reduced neutrophils together with reduced TNF-α may not be a coincidence, since a vast body of research links TNF-α and neutrophil recruitment to inflammation sites [41,42,43,44]. TNF-α promotes recruitment, activation, and prolongs the lifespan of neutrophils at sites of inflammation such as atherosclerotic injuries [43]. We also observed significant reductions in the expressions of E-selectin and ICAM adhesion molecules in the aortas of the CETP-expressing BM reconstituted mice. Selectins are important for leukocyte adhesion, especially neutrophils, as previously reported in studies with mice deficient in E-selectin and P-selectin [45,46,47]. The reduction in the TNF-α (protein) levels in the aorta roots of both the recipient young males and aged females was not paralleled with the TNF-α mRNA levels found in the thoracic aorta segments of the young males. Since atherosclerotic plaque development varies in different vascular locations, it is not surprising that the thoracic aorta gene expression profile is not exactly the same of that observed in the aorta root, a known predilection site for atherogenesis [48]. It is expected that thoracic segments present a major delay (and low susceptibility) in atherosclerotic events as compared to the aorta root and arch [49,50]. Thus, the present data showed earlier atherosclerosis effects, such as the expression of adhesion molecules, but no late signals of unresolved inflammation, such as local TNF-α, in the thoracic segments of the aortas.

The data presented here are in line with a previous study in another mouse model expressing apoE3Leiden/CETP treated with Torcetrapib [51]. The authors showed that CETP inhibition by Torcetrapib enhanced monocyte recruitment and the expression of MCP-1, yielding lesions with accentuated inflammatory properties, as evidenced by increased macrophages and a reduced collagen content [51]. This observation reinforces the anti-inflammatory role of CETP. 

Our recent study showed that macrophages from CETP transgenic mice exhibit attenuated pro-inflammatory gene expression (TNF-α, IL-6, and iNOS), a diminished reactive oxygen species production, a reduced cholesterol accumulation, and phagocytosis [52]. These macrophage-localized functions of CETP may explain, at least in part, the mechanisms underlying the less inflammatory type of atherosclerotic lesions described in the mice that received CETP^+^ BM. In agreement, other studies have reported lower plasma concentrations of TNF-α and IL-6 in transgenic mice expressing CETP compared to wild-types following LPS stimulation or sepsis [16]. Given that inflammation plays a key role in the initiation and progression of atherosclerosis, we may conclude that CETP, in the context of preserved *Nnt* expression, contributes to the development of a less detrimental and more stable type of atherosclerotic lesion.

Although CETP-expressing macrophage features [52] may partially explain the differences in lesion phenotypes, other mechanisms, particularly related to neutrophil accumulation in plaques, were lacking in this study. For example, whether CETP modulates the recruitment, proliferation, and/or viability of neutrophils in plaques deserves further investigation. CETP seems not to promote inflammation resolution, since CETP expression decreases macrophage phagocytic activity [52]. Thus, the ongoing hypothesis is that CETP inhibits inflammation.

Altogether, these data add to the current knowledge as follows: (1) the impact of CETP expression in BM-derived cells on atherogenesis clearly depends on genetic background, especially regarding the redox and inflammatory context, and (2) CETP expression in BM-derived cells, with preserved expressions of *Ldlr* and *Nnt* genes, contributes to reducing the inflammatory features of atherosclerotic lesions.

## Figures and Tables

**Table 1:** Plasma lipids and body and tissue masses of LDL receptor knockout (*Ldlr*^−/−^) mice transplanted with bone marrow of CETP-expressing (CETP^+^) or non-expressing (CETP^0^) mice.

Bone Marrow Recipient	Bone Marrow Donor	
	Cetp^0^	Cetp^+^
Young male *Ldlr*^−/−^		
Plasma triglycerides (mg/dL)	197.7 ± 13.5	178.2 ± 13.8
Plasma cholesterol (mg/dL)	430.5 ± 69.87	614.1 ± 46.25 *
Body weight (g)	25.34 ± 0.65	23.69 ± 0.55
Epididymal fat (%BW)	2.99 ± 0.25	1.79 ± 0.09 *
Subcutaneous fat (%BW)	1.07 ± 0.13	0.73 ± 0.08 *
Brown fat (%BW)	0.28 ± 0.01	0.25 ± 0.009
Liver (%BW)	4.16 ± 0.15	4.23 ± 0.11
Aged female *Ldlr*^−/−^		
Plasma triglycerides (mg/dL)	148.6 ± 18.1	193.8 ± 27.6
Plasma cholesterol (mg/dL)	912.4 ± 131.3	849.3 ± 85.83
Body weight (g)	23.44 ± 0.66	23.09 ± 0.48
Perigonadal fat (%BW)	1.78 ± 0.27	1.89 ± 0.25
Subcutaneous fat (%BW)	0.89 ± 0.11	0.93 ± 0.13
Brown fat (%BW)	0.27 ± 0.01	0.27 ± 0.006
Liver (%BW)	4.07 ± 0.02	4.02 ± 0.05

Recipient mice were fed with a high-fat and -cholesterol diet for 8 weeks. Data are mean ± SEM, n = 12 for both groups of young male and n = 7 for aged female recipient of CETP^0^ bone marrow and n = 11 for aged female recipient of CETP^+^ bone marrow. * *p* < 0.05. Student’s *t* test.

## Data Availability

The data that support the findings of this study are available from the corresponding author upon reasonable request.

## Supplementary Materials

The following supporting information can be downloaded at: https://www.mdpi.com/article/10.3390/biom13101556/s1, Table S1: High fat and high cholesterol diet composition; Table S2: Primer sequences used for RT-PCR.
