# Supplementary material for: CETP Expression in Bone-Marrow-Derived Cells Reduces the Inflammatory Features of Atherosclerosis in Hypercholesterolemic Mice
Source: Biomolecules. 2023 Oct 22;13(10):1556. doi: 10.3390/biom13101556 (PMC10605246; doi:10.3390/biom13101556)
Supplement: Supplementary file 1 [file biomolecules-13-01556-s001.zip › biomolecules-2621251-supplementary.pdf]

Supplementary Material

Table S1. High fat and high cholesterol diet composition

Product	Grams %	Kcal %
Protein	20	17
Carbohydrate	50	43
Fat	21	41
	(4.7 Kcal/g)	100
**Ingredients**	**Grams**	**Kcal**
Casein, 80 Mesh	195	780
DL-Methionine	3	12
Corn Starch	50	200
Maltodextrin 10	100	400
Sucrose	341	1364
Cellulose	50	0
Milk Fat, Anhydrous*	200	1800
Corn Oil	100	90
Mineral Mix S10001	35	0
Calcium Carbonate	4	0
Vitamin Mix V10001	10	40
Choline Bitartrate	2	0
Cholesterol, USP*	1	0
Ethoxyquin	0.04	0
Total	1001.54	4686

*Anhydrous milk fat typically contains approximately 0.3% cholesterol.

Table S2. Primers' sequences used for RT-PCR:

Gene	Primers	
**36B4***	forward	GAGGAATCAGATGAGGATATGGGA
	reverse	AAGCAGGCTGACTTGGTTGC
**ARG1**	forward	GGCGCATTCACAGTCACTTA
	reverse	TGAACCCAACTCTTGGGAAG
**CCR2**	forward	GCCATCATAAAGGAGCCATACC
	reverse	ATGCCGTGGATGAACTGAGG
**CD36**	forward	GGAACTGTGGGCTCATTGC
	reverse	CATGAGAATGCCTCCAAACAC
**CDC42**	forward	ACGTGTCCCCACCTGGTGCT
	reverse	GCGAGACAGCGTCCACCCAC
**CETP** (simian)	forward	CAAATCAGCCACTTGTCCAT
	reverse	CAGTTGTGTGTTGATCTGGA
**ICAM1**	forward	GTGATGCTCAGGTATCCATCCA

	reverse	CACAGTTCTCAAAGCACAGCG
**IL-10**	forward	GCTCTTACTGACTGGCATGAG
	reverse	CGCAGCTCTAGGAGCATGTG
**IL-1β**	forward	CCTTCCAGGATGAGGACATGA
	reverse	TGAGTCACAGAGGATGGGCTC
**IL-4**	forward	CCAAACGTCCTCACAGCAAC
	reverse	AAGCCCGAAAGAGTCTCTGC
**IL-6**	forward	CACGGCCTTCCCTACTTCAC
	reverse	GGTCTGTTGGGAGTGGTATC
**iNOS**	forward	GTTCTCAGCCCAACAATACAAGA
	reverse	GTGGACGGGTCGATGTCAC
**PLXND1**	forward	TCGCTGCCAATCCCTAATAAGA
	reverse	TGACCTGGTTTGGAACTGTTG
**SELE**	forward	ATGCCTCGCGCTTTCTCTC
	reverse	GTAGTCCCGCTGACAGTATGC
**TLR4**	forward	CCATGCATTTGGCCTTAGCC
	reverse	AGAGCACTGAACCTCCTTGC
**TNF-α**	forward	CCCTCCTGGCCAACGGCATG
	reverse	TCGGGGCAGCCTTGTCCCTT
**VCAM**	forward	TAGAGTGCAAGGAGTTCGGG
	reverse	CCGGCATATACGAGTGTGAA

*acidic ribosomal phosphoprotein P0 (RPLP0). All primers are 5′→3′
